# Identification of the predominant nonrestoring allele for Owen-type cytoplasmic male sterility in sugar beet (*Beta vulgaris* L.): development of molecular markers for the maintainer genotype

**DOI:** 10.1007/s11032-013-9854-8

**Published:** 2013-03-14

**Authors:** Mari Moritani, Kazunori Taguchi, Kazuyoshi Kitazaki, Hiroaki Matsuhira, Takaya Katsuyama, Tetsuo Mikami, Tomohiko Kubo

**Affiliations:** 1 Research Faculty of Agriculture, Hokkaido University, N-9, W-9, Kita-ku, Sapporo, 060-8589 Japan; 2 Memuro Upland Farming Research Station, Hokkaido Agricultural Research Center (HARC), National Agriculture and Food Research Organization (NARO), Memuro, Hokkaido 082-0081 Japan; 3 Present Address: HARC-NARO, Sapporo, Hokkaido 062-8555 Japan; 4 Present Address: Central Research Institute of Electric Power Industry, Abiko, Chiba 270-1194 Japan

**Keywords:** Marker-assisted selection, Restorer of fertility, Genetic association, PCR marker, Multi-allelic locus

## Abstract

Hybrid seed production in sugar beet relies on cytoplasmic male sterility (CMS). As time-consuming and laborious test crosses with a CMS tester are necessary to identify maintainer lines, development of a marker-assisted selection method for the *rf* gene (the nonrestoring allele of *restorer*-*of*-*fertility* locus) is highly desirable for sugar-beet breeding. To develop such a method, we investigated genetic variation at the *Rf1* locus, one of two *Rf* loci known in sugar beet. After *Hin*dIII-digestion, genomic DNAs from beet plants known to have a restoring *Rf1* allele yielded a range of hybridization patterns on agarose gels, indicating that *Rf1* is a multi-allelic locus. However, the hybridization patterns of 22 of 23 maintainer lines were indistinguishable. The nucleotide sequences of the *rf1* coding regions of these 22 maintainer lines were found to be identical, confirming that the lines had the same *rf1* allele. Two PCR markers were developed that targeted a downstream intergenic sequence and an intron of *Rf1.* The electrophoretic patterns of both markers indicated multiple *Rf1* alleles, one of which, named the dd(L) type, was associated with the maintainer genotype. To test the validity of marker-assisted selection, 147 sugar beet plants were genotyped using these markers. Additionally, the 147 sugar beet plants were crossed with CMS plants to determine whether they possessed the maintainer genotype. Analysis of 5038 F1 offspring showed that 53 % of the dd(L) plants, but none of the plants with other alleles, had the maintainer genotype. Thus, selection for the dd(L) type considerably enriched the proportion of plants with the maintainer genotype.

**Electronic supplementary material:**

The online version of this article (doi:10.1007/s11032-013-9854-8) contains supplementary material, which is available to authorized users.

## Introduction

Cytoplasmic male sterility (CMS) is an invaluable character for hybrid seed production in many crop species as it can be used to provide seed parents without the need for an emasculation procedure (Schnable and Wise 1998; Hanson and Bentolila 2004; Budar et al. 2006; Chase 2007; Kubo et al. 2011). CMS in sugar beet (*Beta vulgaris* L.) was first described by Owen (1942, 1945) who observed that male sterility resulted from the combined action of at least two nuclear genes, termed *X* and *Z* (now termed restorer-of-fertility [*Rf*] genes), and sterilizing cytoplasm (S). Owen proposed that completely male-sterile plants have the genotype [S]*xxzz*, with the other genotype combinations ([S]*XXZZ*, [S]*XXZz*, [S]*XXzz*, [S]*XxZZ*, [S]*XxZz*, [S]*Xxzz*, [S]*xxZZ*, and [S]*xxZz*) usually showing a varying degree of pollen fertility (Bosemark 2006).

Propagation of CMS plants requires an equivalent pollen-fertile maintainer genotype (referred to as an O-type in sugar beet) that has normal (fertile) cytoplasm (N) and is recessive at the same two loci ([N]*xxzz*); such maintainer plants owe their pollen production ability to the N cytoplasm, in contrast to the S cytoplasm (Owen 1945). Monogenic pollen fertility restoration has been reported in sugar beet (Theurer 1971; Pillen et al. 1993). In addition to these major genes, phenotypic expression may be affected by modifying gene(s) (Hogaboam 1957). There is no doubt that the inheritance of fertility restoration in Owen cytoplasm is more complicated than originally hypothesized (see Hjerdin-Panagopoulos et al. 2002). Nevertheless, strict selection for the maintainer genotype enables the identification of maintainer lines to produce stable CMS lines (Nielson and Nemazi 1967; Bosemark 1972).

Sugar beet plants with the maintainer genotype are comparatively rare (less than 5 %) in most beet populations (Bosemark 2006). When developing new maintainer lines using beet germplasm accessions, the only currently available method for breeders to determine definitively the genotype of a given plant is to cross it to a CMS tester line and assess the male sterility/fertility of the resulting F1 hybrids. Marker-assisted selection (MAS) would eliminate the need for such laborious test crosses.

We previously carried out inheritance studies involving a sugar beet restorer line, NK-198, and identified a monogenic restoration pattern (Hagihara et al. 2005). The gene was named *Rf1* and appeared to be indistinguishable from the *X* gene, based on its map position (Hagihara et al. 2005). BAC clones covering the *Rf1* region (a total of 383 kbp) were sequenced to reveal quadruplicated open reading frames (ORFs) (termed *bvORF18*, *bvORF19*, *bvORF20*, and *bvORF21*) with significant homology to yeast *Oma1* metalloprotease (Matsuhira et al. 2012). Most significantly, *bvORF20* restored male fertility to CMS sugar beet when expressed as a transgene, whereas the other three ORFs did not (Matsuhira et al. 2012). In contrast, the corresponding region of a maintainer line, TK-81mm-O, only contained a single ORF with 85 % identity to *bvORF20*; this ORF was named *bvORF20L*. These results indicate that *bvORF20* represents an *Rf1* restoring allele and that *bvORF20L* represents an *rf1* nonrestoring allele. However, the question arises whether *bvORF20* and *bvORF20L* are common alleles in most restorer and maintainer lines, respectively.

In this study, we show that *Rf1* is a multi-allelic locus and that the TK-81mm-O-type *bvORF20L* is predominant in Japanese maintainer lines. We also report the development and use of molecular markers based on the sequence data from the intronic and flanking regions of *bvORF20L*. Our analyses indicate that these markers can enrich maintainer genotypes in a broad range of beet germplasms.

## Materials and methods

### Plant materials

Plant materials used in this study are listed in Table 1. NK-198, NK-305, and NK-322 are restorer lines developed by Hokkaido Agricultural Research Center (HARC), National Agriculture and Food Research Organization (NARO), Japan. Sugar beet cultivar (cv.) Donyu-2 is a selection from the open-pollinated cv. GW359, initially developed by the Great Western Sugar Company (GW), USA. Donyu-2 was released by the Japan Sugar Beet Improvement Foundation (succeeded by HARC-NARO) in 1954. Tenken-1 is a synthetic variety released in 1964, produced by intercrossing US401 (a US cultivar), AJ4 (a Polish cultivar), and Donyu-2. TA-26 (developed in Germany), TA-36 (Germany), TA-48 (Canada), TA-49 (Canada), TA-226 (Denmark), and TA-541 (Hungary) are varieties that were introduced before the 1960s by the Japan Sugar Beet Improvement Foundation. I-12 61L, I-12CMS(R), I-12CMS(2), and I-12CMS(3) were developed by GW. I-12CMS(R) has the Owen cytoplasm, and I-12CMS(2) and I-12CMS(3) possess sterilizing cytoplasm derived from wild beets collected in Turkey and Pakistan, respectively (Mikami et al. 1985; Yamamoto et al. 2008). I-12 61L can be used as a common maintainer for these three CMS lines. The other lines (NK-169mm-O through TK-81mm-O) were developed by HARC, in which those with an ‘-O’ suffix are maintainer lines. TK-76mm-CMS is an Owen-type CMS line maintained by TK-76mm-O. Crosses were made by exchanging paper bags over the inflorescences in the greenhouse. Pollen-producing seed parents were emasculated and bagged to prevent pollen contamination. Sib matings were made from the F2 through to the F4 generation.

**Table 1:** Plant materials used in this study and their marker patterns

Cultivar	Nuclear genotype	Source	Marker type and number of plants															
			aa(S)	ab(LS)	bb(LS)	bb(S)	bc(LS)	bc(S)	bd(LS)	bd(S)	cc(LS)	cc(S)	cd(LS)	cd(S)	dd(L)	dd(LS)	dd(S)	Total
NK-198	Restorer	HARC	1															1
NK-305	Restorer	HARC				25												25
NK-322	Restorer	HARC																ND
Donyu-2	Unknown	HARC			1	1		1										3
Tenken-1	Unknown	HARC																ND
TA-36	Unknown	HARC			16	68	1	7	3	12		4	1	1	5		1	119
TA-48	Unknown	HARC																ND
TA-49	Unknown	HARC																ND
TA-226	Unknown	HARC		1	10		9		17		4		2		1			44
TA-541	Unknown	HARC			11		10		6		7							34
I-12 61L	Maintainer	GW													2			2
I-12CMS(2)	Maintainer	GW													2			2
I-12CMS(3)	Maintainer	GW													1			1
I-12CMS(R)	Maintainer	GW													2			2
NK-169mm-O	Maintainer	HARC													28			28
NK-172BRmm-O	Maintainer	HARC							8						12			20
NK-183BRmm-O	Maintainer	HARC													4			4
NK-184mm-O	Maintainer	HARC																ND
NK-185BRmm-O	Maintainer	HARC																ND
NK-204mm-O	Maintainer	HARC													4			4
NK-205mm-O	Maintainer	HARC													4			4
NK-206mm-O	Maintainer	HARC													6			6
NK-208mm-O	Maintainer	HARC										4			1			5
NK-219mm-O	Maintainer	HARC			47													47
NK-222BRmm-O	Maintainer	HARC			4		5		9						2			20
NK-226BRmm-O	Maintainer	HARC													7			7
NK-252mm-O	Maintainer	HARC													7			7
NK-280mm-O	Maintainer	HARC													10			10
NK-294mm-O	Maintainer	HARC													8			8
NK-296mm-O	Maintainer	HARC													20			20
NK-300mm-O	Maintainer	HARC							1						19			20
NK-310mm-O	Maintainer	HARC													27			27
NK-325mm-O	Maintainer	HARC											1		19			20
NK-341mm-O	Maintainer	HARC			1				8						11			20
NK-343mm-O	Maintainer	HARC							5						15			20
TA-33BB-O	Maintainer	HARC													11			11
TK-76mm-CMS	Maintainer	HARC													2			2
TK-76mm-O	Maintainer	HARC													9			9
TK-81mm-O	Maintainer	HARC										2	22		21			45

*ND* no data

### Male fertility phenotyping

Individual plants were carefully evaluated for anther color, dehiscence and pollen production during the flowering period. Classification of male-sterile plants is shown in Table 2 (modified from Imanishi et al. 1970).

**Table 2:** Classification of male steriles (modified from Imanishi et al. 1970)

Class	Characteristics of anther		
	Color	Dehiscence	Pollen production
W	White or brown	−	−
G	Light green	−	−
S	Yellow, sometimes orange	−, rarely +	−
P	Yellow, sometimes orange	Both + and − are seen	±
N	Yellow	+	+

### DNA isolation

Total cellular DNA was isolated using the method of Doyle and Doyle (1990). The sample DNA for gel blot analysis was further purified by centrifugation in CsCl–EtBr continuous density gradients (Sambrook et al. 1989).

### DNA gel blot analysis

Five μg of total cellular DNA was digested with *Hin*dIII restriction endonuclease purchased from Takara Bio (Ohtsu, Japan). The samples were electrophoresed on 1 % agarose gels. DNA transfer to Hybond N+ membrane was performed according to the manufacturer’s instructions (GE Healthcare UK, Amersham Place, England). DNA fragments of interest were labeled with alkaline phosphatase using the AlkPhos Direct DNA labeling system (GE Healthcare UK).

### Polymerase chain reaction (PCR) and cleaved amplified polymorphic sequence (CAPS) detection

The nucleotide sequences of the primers used in this study are listed in Table S1. The 17-20L sequence was amplified in a 20-μL solution containing 0.4 U of LA-Taq (Takara Bio), 2.5 mM of MgCl_2_, 0.25 mM of each dNTP, 0.2 μM of each primer, and ~10 ng of genomic DNA with the 1× buffer supplied by the manufacturer. The reaction mixture without MgCl_2_ was pre-heated in a thermal cycler followed by addition of the MgCl_2_ solution supplied by the manufacturer. The PCR protocol was 94 °C for 3 min, 35 cycles of 94 °C for 30 s and 68 °C for 5 min plus 10 s per cycle, and 72 °C for 10 min. PCR products were digested with *Hap*II (Takara Bio) in a 20-μL solution containing 10 μL of the resultant PCR solution with the manufacturer’s recommended buffer concentration. Amplification of the 20L-int sequence was performed in a 10-μL solution containing 0.2 μM of each primer, ~5 ng of genomic DNA, and 5 μL of GoTaq (Promega, Madison, WI, USA). The PCR protocol was 35 cycles of 94 °C for 3 s, 58 °C for 1 min and 72 °C for 1 min. The DNA fragment used for preparation of the DNA probe was PCR amplified using the primers shown in Table S1 with plasmid DNA bearing the sequence of interest as the template.

## Results

### *Rf1* gene variation in sugar beet

**Fig. 1:**
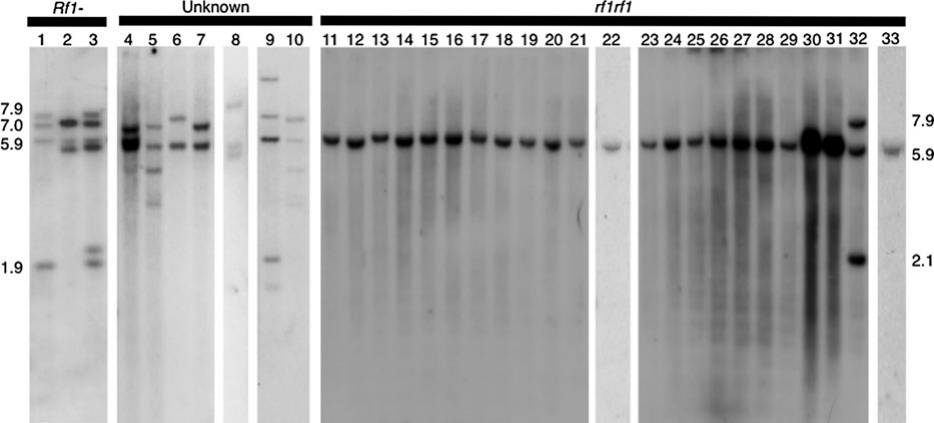
DNA gel blot analysis of the conserved 3′ UTR of *Rf1* (represented by *bvORF19* of NK-198) hybridized with total cellular DNA from various sugar beets. Band sizes are shown in kbp. Genotype at the *Rf1* locus is indicated if known. *1* NK-198, *2* NK-305, *3* NK-322, *4*–*7* individuals of Tenken-1, *8*–*10* individuals of Donyu-2, *11* NK-206mm-O, *12* NK-169mm-O, *13* NK-300mm-O, *14* NK-204mm-O, *15* NK-280mm-O, *16* NK-208mm-O, *17* TA-33BB-O, *18* NK-294mm-O, *19* NK-226BRmm-O, *20* NK-183mm-O, *21* NK-310mm-O *22* NK-184mm-O, *23* NK-205mm-O, *24* NK-252mm-O, *25* TK-76mm-O, *26* TK-81mm-O, *27* NK-172mm-O, *28* NK-296mm-O, *29* NK-325mm-O, *30* NK-341mm-O, *31* NK-343mm-O, *32* NK-219mm-O, *33* I-12 61L

A comparison of *bvORF18*-*bvORF21* (the quadruplicated ORF in the restorer NK-198 line) and *bvORF20L* (the ortholog in the maintainer TK-81mm-O line) revealed that the 3′ untranslated region (UTR) and downstream sequence (249 bp) of these ORFs share >95 % identity (see Matsuhira et al. 2012). A DNA fragment of 346 bp including the 3′ UTR and its surrounding sequence was used as a probe on Southern blots of total DNAs from various sugar beet lines in order to analyze organizational variation of the *Rf1* locus. As shown in Fig. 1, four *Hin*dIII fragments (7.9, 7.0, 5.9, and 1.9 kbp) were observed for NK-198. Two other restorer lines, NK-305 and NK-322, had a different pattern, although they had the 7.0- and 5.9-kbp fragments in common with NK-198. Four plants of an old synthetic variety, Tenken-1, had inter-individual differences in their hybridization patterns and also differences compared to the restorer line. Similar intra-cultivar heterogeneity was also noted in the open-pollinated cultivar Donyu-2. One Donyu-2 plant had a similar band pattern to one of the Tenken-1 plants (compare lanes 5 and 10 in Fig. 1). This result is not unexpected because Donyu-2 was used as a constituent line in developing Tenken-1. Overall, the analysis indicates that there is a substantial degree of variation including copy number variation in the organization of *Rf1* and its related genes clustered in the *Rf1* locus.

### *bvORF20L* is the most frequent non-restoring *rf1* allele in Japanese maintainer lines

A 5.9-kbp hybridization signal from the *bvORF20L* sequence was detected in *Hin*dIII-digested DNA from TK-81mm-O plants (Fig. 1). This signal was shared by 21 Japanese maintainer lines and an American maintainer line; however, the signal was not present in the Japanese maintainer line NK-219mm-O, which instead had 7.9-, 5.9-, and 2.1-kbp *Hin*dIII fragments (Fig. 1).

According to the Southern blot analysis, the frequency of NK-219mm-O *rf1* was apparently low in Japanese maintainer lines, and we had no other line in which the organization of *rf1* was similar to NK-219mm-O-*rf1*. On the other hand, TK-81mm-O-like *rf1* occurred frequently in Japanese maintainer lines, and we questioned whether these *rf1* were identical to TK-81mm-O *rf1*. The *bvORF20L* copies from the 21 Japanese maintainer lines were PCR amplified with primers 20L-Fw and 20L-Rv and directly sequenced. The nucleotide sequences from all the lines were identical and matched the sequence in the TK-81mm-O line, indicating that a large number of Japanese maintainer lines have the same *rf1* nonrestoring allele in common. Hereafter, we focused our analysis on TK-81mm-O *rf1*.

### Development of markers 17-20L and 20L-int

**Fig. 2:**
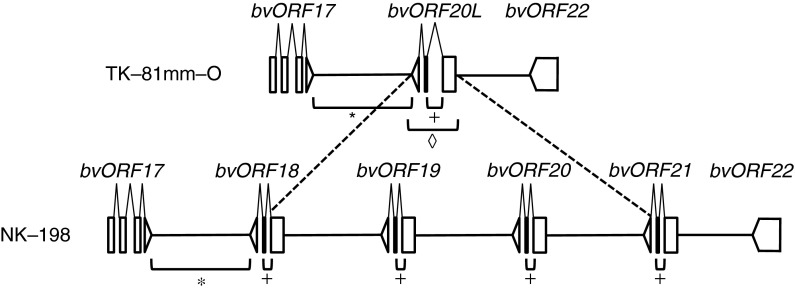
Schematic illustration of target regions of 17-20L and 20L-int markers in TK-81mm-O and NK-198 (adapted from Matsuhira et al. 2012). *Open boxes* indicate the exons and *wedges* indicate the introns. NK-198-homologs of *bvORF20L* are quadruplicated as *bvORF18*, *bvORF19*, *bvORF20*, and *bvORF21*. Target sequences of 17-20L (PCR amplified by primers 17-20L-Fw and 17-20L-Rv), 20L-int (by primers 20L-int-Fw and 20L-int-Rv), and entire *bvORF20L* amplification (by 20L-Fw and 20L-Rv) are shown by *brackets* with *asterisks*, *plus signs*, and a *diamond*, respectively

We compared the two nucleotide sequences, one containing the quadruplicated ORFs found in the *Rf1* locus of the NK-198 restorer line (DDBJ/EMBL/GenBank accession numbers AB646133 and AB646135) and the other containing *bvORF20L* from TK-81mm-O (AB646136) (Fig. 2). This comparison indicated that two regions of the NK-198 and TK-81mm-O loci might be useful for marker development. The development and validation of these two potential diagnostic markers (named 17-20L and 20L-int) are described in detail below.

**Fig. 3:**
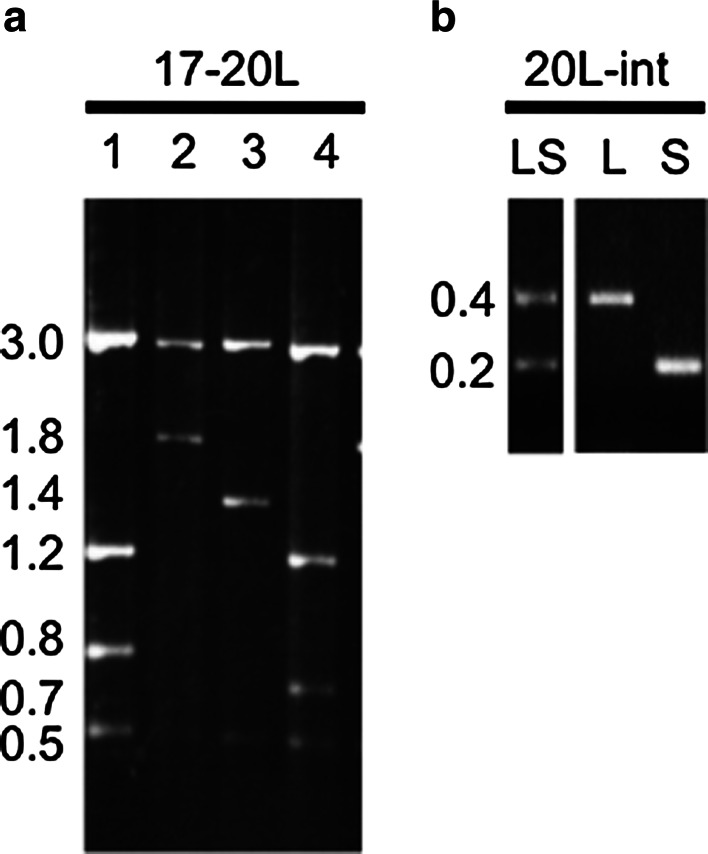
Agarose gel electrophoresis of 17-20L and 20L-int markers. **a** Four typical band patterns produced using the 17-20L marker. *Lanes 1*–*4* correspond to patterns ‘a’ to ‘d’, respectively. Size markers are shown on the *left* (kpb). **b** Three typical band patterns produced using the 20L-int marker. Size markers are shown on the *left* (kbp)

17-20L is a cleaved amplified polymorphic sequence (CAPS) marker derived from the *bvORF17*-*bvORF20L* intergenic region. The primer combination 17-20L-Fw/17-20L-Rv generated a ~5.5-kbp amplicon in all beet plants examined in this study. Digestion of this PCR amplicon with *Hap*II, however, yielded four polymorphic electrophoretic patterns. Two fragments of 3.0 and 0.5 kbp were present in all four patterns in addition to polymorphic fragments: pattern ‘a’ contained 1.2- and 0.8-kbp fragments; pattern ‘b’ contained a 1.8-kbp fragment; pattern ‘c’ contained a 1.4-kbp fragment; and pattern ‘d’ contained 1.2- and 0.7-kbp fragments (Fig. 3a). Since the 17-20L targeted sequence was regarded as a single copy (see Fig. 2), this marker enables genotyping for the 17-20L locus.

20L-int is derived from the first intron of *bvORF20L*. The intron differed in length between *bvORF20L* and the quadruplicated ORFs. The intron of *bvORF20L* was 352 bp compared to 142–167 bp for the quadruplicated ORFs. In all the plants examined, the 20L-int-Fw and 20L-int-Rv primers amplified fragments that fell into two classes: 0.4-kbp (class L) and ~0.2-kbp (class S) amplicons (Fig. 3b). A 0.4-kbp band was expected from amplification of *bvORF20L*; however, the ~0.2-kbp band appeared to be a mixture of fragments that differed slightly in size (data not shown). As the target sequence of 20L-int was not always a single copy, such as in the quadruplicated ORFs of NK-198 (see Fig. 2), we were not able to determine genotypes at the 20L-int locus. Nevertheless, the 0.4-kbp band could be used as a diagnostic marker for the presence of the L intron and the ~0.2-kbp band as a marker for the presence of the S intron.

### A predominant marker type in Japanese maintainer lines

To be of value in a breeding program, a marker should be able to identify polymorphisms among different genetic backgrounds; this characteristic is referred to as marker validation. We therefore screened a range of beet genotypes with 17-20L and 20L-int to determine their utility as markers. First, we tested three of the Donyu-2 plants that were used in the Southern analysis (see Fig. 1). One plant (lane 8 in Fig. 1) had a b/b band profile pattern for the 17-20L locus, and an S intron band profile for the 20L-int locus [hereafter called bb(S)]. The other two plants (lanes 9 and 10) were respectively scored as bc(S) and bb(LS). The bb(S), bc(S), and bb(LS) marker patterns also occurred frequently in three other cultivars (Table 1). Twelve additional marker types were identified in sugar beet cultivars, indicating a high degree of genetic variation at the two loci in these germplasm accessions (Table 1).

A beet plant that is homozygous for TK-81mm-O *bvORF20L* is expected to show a dd(L) marker pattern; that is, the d/d band profile pattern for 17-20L and the L intron band profile for 20L-int. Beet plants of 21 Japanese maintainer lines that carry TK-81mm-O *rf1* (see Fig. 1) were tested using the 17-20L and 20L-int markers. Total cellular DNAs from these plants were used as templates for a PCR analysis. All the plants were scored as dd(L) (data not shown).

The sugar beet genotypes surveyed here consisted mostly of CMS lines (four lines) and maintainer lines (23 lines) that are presumed to have the nuclear genotype *xxzz* (*rf1rf1rf2rf2*). In total, 371 plants from these lines were typed for the 17-20L and 20L-int markers and 254 (68 %) were scored as dd(L). The remaining 117 plants were assigned to five marker types: bb(LS), 52 plants; bd(LS), 31 plants; cd(LS), 23 plants; cc(S), six plants; and bc(LS), five plants (Table 1). Notably, 14 Japanese lines, e.g. NK-169mm-O, and four American lines, e.g. I-12 61L, showed only the dd(L) marker pattern (Table 1), although the number of plants tested per line was limited. In contrast, the dd(L) marker pattern was rare in three maintainer lines, NK-208mm-O, NK-219mm-O, and NK-222BRmm-O (Table 1).

### Testing the 17-20L and 20L-int markers for selection of maintainer genotypes

To test whether selection of dd(L) type plants is indeed effective for identifying maintainer genotypes, the 17-20L and 20L-int markers were used to screen F4 plants derived from three cross combinations, NK-280mm-O × TA-49, TA-48 × NK-185BRmm-O, and TA-26 × NK-185BRmm-O. The dd(L) type plants, along with several segregants with other marker types, namely, bb(S), bc(S), and bd(S), were selected and used as pollen parents in crosses with the annual Owen-CMS tester line, TA-33BB-CMS. TA-33BB-CMS has been maintained by a maintainer line TA-33BB-O, scored as dd(L) (see Table 1). Our test crosses also included 69 plants of various marker types that were selected from an old cultivar, TA-36. A minimum of 10 progeny from each cross (5038 plants in total) were grown in the greenhouse and examined for male sterility. For example, a dd(L) plant (designated B7) from F4 (NK-280mm-O × TA-49), when crossed to TA-33BB-CMS, resulted in 100 % male sterile offspring, indicating that B7 had the maintainer genotype (Table S2). In contrast, a bb(S) plant (designated B8) from the same cross produced no offspring that was classified as completely male-sterile (anther phenotype ‘W’). The numbers of pollen parents that behaved as maintainer [defined as those producing >95 % completely male-sterile plants (Class W) in F1 offspring] or near-maintainer [90–95 % of completely male-sterile plants (Class W) in F1 offspring] are summarized in Table 3. No fully male-fertile offspring (Class N) were observed in the test cross progenies of dd(L) plants (see Table S2). We found that the cross with dd(L) plants from F4 (NK-280mm-O × TA-49) had a high rate of perfect male-steriles (between 92 and 100 %), and all the 12 dd(L) plants were either maintainer or near-maintainer genotypes (Table 3). Most of the dd(L) plants from F4 (TA-26 × NK-185BRmm-O) were also classified as either maintainer or near-maintainer genotypes (~90 %). On the other hand, only 4.7 % of the dd(L) plants from F4 (TA-48 × NK-185BRmm-O) were judged to be maintainer genotypes. One dd(L) plant from TA-36 was near-maintainer genotype (Table 3).

**Table 3:** Summary of 17-20L and 20L-int marker patterns and results of test crosses

Plants selected as pollen parent		Number of plants classified as			Total
Population	Marker type	Maintainer^a^	Near-maintainer^b^	Others	
TA-36	bb(LS)	0	0	5	5
	bb(S)	0	0	36	36
	bc(LS)	0	0	1	1
	bc(S)	0	0	7	7
	bd(LS)	0	0	2	2
	bd(S)	0	0	8	8
	cc(S)	0	0	4	4
	cd(LS)	0	0	1	1
	cd(S)	0	0	1	1
	dd(L)	0	1	3	4
F4 (NK-280mm-O × TA-49)	bb(S)	0	0	8	8
	dd(L)	10	2	0	12
F4 (TA-48 × NK-185BRmm-O)	bc(S)	0	0	1	1
	bd(S)	0	0	1	1
	dd(L)	1	0	20	21
F4 (TA-26 × NK-185BRmm-O)	bb(S)	0	0	2	2
	bd(S)	0	0	4	4
	dd(L)	24	2	3	29

^a^Percentage of completely male-sterile plants (Class W) in the F1 of the selected pollen parent plant × TA-33BB-CMS: >95 %

^b^Percentage of completely male-sterile plants (Class W) in the F1 of the selected pollen parent plant × TA-33BB-CMS: 90–95 %

By contrast, all plants with marker patterns other than dd(L) were identified as non-maintainer genotypes (‘Others’ in Table 3). These plants, when crossed with TA-33BB-CMS, yielded fully and partially male-fertile offspring as well as male-sterile offspring. Thus, selection of the dd(L) plants clearly resulted in an increase in the frequency of the maintainer genotype among the breeding materials.

## Discussion

A prerequisite for successful marker-assisted selection of sugar beet maintainer genotypes is identifying variation in the organization of the *Rf1* locus. Our DNA gel blot analysis with the *Rf1* probe revealed polymorphic band patterns among the sugar beet germplasm accessions tested, suggesting that *Rf1* is a complex, multi-allelic locus. The RFLPs cannot be used to characterize the restoring alleles carried by the restorer lines NK-305 and NK-322, but 22 Japanese maintainer lines all showed the same band pattern and could be characterized as having the same *rf1* nonrestoring allele. Nucleotide sequence analysis confirmed that the nucleotide sequences of the *rf1* allele of 22 maintainer lines were 100 % identical. This result could be due to a genetic bottleneck because Japanese sugar beet lines were selected from a small number of lines/cultivars developed outside of Japan (Taguchi et al. 2006). Furthermore, the repeated use of the same CMS tester lines in maintainer development and selection processes in Japan may have significantly restricted the number of nonrestoring *rf1* alleles.

This study has resulted in the development of two agarose gel-based and user-friendly markers (17-20L and 20L-int) for selecting a maintainer genotype for Owen CMS in sugar beet. 17-20L is a CAPS marker and is easy to handle. The informative *Hap*II restriction fragments are relatively long (0.7–1.8 kbp) ensuring that they can be resolved on standard 1.5–2 % agarose gels. In addition, the presence of several restriction fragments reduces the risk of misidentification. The other marker, 20L-int, originated from the first intron of *bvORF20L* and can differentiate two intronic size classes (L and S). By combining data for the 17-20L and 20L-int loci, we were able to distinguish 15 marker patterns in a range of cultivated beet accessions. Of these marker patterns, dd(L) particularly attracted our attention because it showed significant association with *bvORF20L*, an *rf1* variant commonly found in the majority of maintainer lines examined.

We also selected dd(L) plants from three crosses among sugar beet lines as well as plants chosen randomly from an old, heterogeneous cultivar to investigate whether the selected dd(L) plants had the maintainer genotype. Sixty-six dd(L) plants were selected and crossed to an Owen-CMS tester; half of the selected plants had a perfect maintainer genotype. By contrast, a maintainer genotype was never observed in plants with marker patterns other than dd(L), clearly indicating that selection of the dd(L) pattern is effective.

The marker system developed here will allow a simple and rapid assay of the maintainer genotype in a given beet population, although test crosses will be still necessary for final confirmation. Pre-selection by the two DNA markers can reduce the scale of maintainer selection since screening can be undertaken as early as the seedling stage. Thus, the use of this new marker system will greatly reduce the labor required for breeding programs in this crop species. One limitation of the method is that the marker system is not always efficient in identifying perfect maintainer plants. As can be seen in Table 3, whereas dd(L) selection was very efficient in identifying maintainers from F4 (NK-280mm-O × TA-49) and F4 (TA-26 × NK-185BRmm-O), 24 of 25 dd(L) plants of TA-36 and F4 (TA-48 × NK-185BRmm-O) produced partially male-sterile progeny when crossed to the Owen-CMS tester (Table S2). This observation is not surprising because the *Rf1* (*X*) locus likely exerts a major influence on fertility but is by no means the only locus involved in fertility restoration in Owen-CMS (Bosemark 2006). A second locus, *Z,* is known to interact with *Rf1* to fully restore fertility (Owen 1950).

Originally, Owen (1945) proposed that the complementary action of the *X* and *Z* loci governs male sterility or fertility, but he also pointed out that this genetic model did not explain all the results of the crosses he made. Later, he proposed that the *Z* locus has a minor influence on fertility compared with the *X* locus (Owen 1950). We are currently constructing a regional linkage map around the *Z* locus, where another restorer gene provisionally termed *Rf2* resides, and we are currently attempting to develop molecular markers that are diagnostic for genotyping this genomic region. Combined selection for *Rf1* and *Rf2* loci would enhance the accuracy of marker-assisted selection.

Notably, dd(L) plants are scarce or absent in three Japanese maintainer lines (see Table 1). For example, NK-219mm-O was found to consist exclusively of bb(LS) plants (see also Fig. 1), although the maintainer status of this line was established and deemed to be sufficient to be a constituent of a hybrid variety (Kuranouchi et al. 1994). This point raises the possibility that there might be other, as yet uncharacterized, allelic forms of *rf1*. Such maintainer lines thus merit further investigation. It is currently uncertain how many *rf1* alleles are present in the various maintainer lines. The number is likely to be small because of the infrequent occurrence of maintainer genotypes in most sugar beet populations (Bosemark 2006). Currently we are investigating variations in the chromosomal segment corresponding to *bvORF20L* in the *B. vulgaris* gene pool.

## Electronic supplementary material

Below is the link to the electronic supplementary material.

Supplementary material 1 (XLS 23 kb)

Supplementary material 2 (XLS 41 kb)

## References

[CR1] Bosemark NO (1972). Studies of cytoplasmic male sterility in sugar beet. Report of an I. I. R. B. joint study. J Int Inst Sugar Beet Res.

[CR2] Bosemark NO, Draycott AP (2006). Genetics and breeding. Sugar beet.

[CR3] Budar F, Touzet P, Pelletier G (2006) Cytoplasmic male sterility. In: Ainsworth C (ed) Flowering and its manipulation. Blackwell, Oxford, pp 147–180

[CR4] Chase CD (2007). Cytoplasmic male sterility: a window to the world of plant mitochondrial–nuclear interactions. Trends Genet.

[CR5] Doyle JJ, Doyle JL (1990). Isolation of plant DNA from fresh tissue. Focus.

[CR6] Hagihara E, Itchoda N, Habu Y, Iida S, Mikami T, Kubo T (2005). Molecular mapping of a fertility restorer gene for Owen cytoplasmic male sterility in sugar beet. Theor Appl Genet.

[CR7] Hanson MR, Bentolila S (2004). Interactions of mitochondrial and nuclear genes that affect male gametophyte development. Plant Cell.

[CR8] Hjerdin-Panagopoulos A, Kraft T, Rading IM, Tuvesson S, Nilsson NO (2002). Three QTL regions for restoration of Owen CMS in sugar beet. Crop Sci.

[CR9] Hogaboam GJ (1957). Factors influencing phenotype expression of cytoplasmic male sterility in the sugar beet. J Am Soc Sugar Beet Technol.

[CR10] Imanishi S, Takeda T, Hosokawa S (1970). Frequency of so-called type O plants as a maintainer of cytoplasmic male sterility in sugar beet populations. Jpn J Breed.

[CR11] Kubo T, Kitazaki K, Matsunaga M, Kagami H, Mikami T (2011). Male sterility-inducing mitochondrial genomes: how do they differ?. Crit Rev Plant Sci.

[CR12] Kuranouchi T, Kawakatsu M, Nakano M, Tanaka M (1994). Breeding of a new sugar beet cultivar “Mighty”. Proc Jpn Soc Sugar Beet Technol.

[CR13] Matsuhira H, Kagami H, Kurata M, Kitazaki K, Matsunaga M (2012). Unusual and typical features of a novel restorer-of-fertility gene of sugar beet (*Beta vulgaris* L.). Genetics.

[CR14] Mikami T, Kishima Y, Sugiura M, Kinoshita T (1985). Organelle genome diversity in sugar-beet with normal and different sources of male sterile cytoplasms. Theor Appl Genet.

[CR15] Nielson K, Nemazi (1967). Selection for the type O character in *Beta vulgaris*. J Am Soc Sugar Beet Technol.

[CR16] Owen FV (1942). Male sterility in sugar beets produced by complementary effects of cytoplasmic and Mendelian inheritance. Am J Bot.

[CR17] Owen FV (1945). Cytoplasmically inherited male-sterility in sugar beets. J Agric Res.

[CR18] Owen FV (1950). The sugar beet breeder’s problem of establishing male-sterile populations for hybridization purposes. Proc Am Soc Sugar Beet Technol.

[CR19] Pillen K, Steinrücken G, Hermann RG, Jung C (1993). An extended linkage map of sugar beet (*Beta vulgaris* L.) including nine putative lethal genes and the restorer gene *X*. Plant Breed.

[CR20] Sambrook J, Fritsch EF, Maniatis T (1989). Molecular cloning: a laboratory manual.

[CR21] Schnable PS, Wise RP (1998). The molecular basis of cytoplasmic male sterility and fertility restoration. Trends Plant Sci.

[CR22] Taguchi K, Nakatsuka K, Takahashi H, Okazaki K, Yoshida T (2006). Relationship between the coefficient of parentage and sugar yield in sugar beet F_1_ hybrid. Breed Res.

[CR23] Theurer JC (1971). Inheritance studies of a pollen restorer from Ruby Queen table beet. J Am Soc Sugar Beet Technol.

[CR24] Yamamoto MP, Shinada H, Onodera Y, Komaki C, Mikami T, Kubo T (2008). A male sterility-associated mitochondrial protein in wild beets causes pollen disruption in transgenic plants. Plant J.

